# Convention of Medical Teachers

**Published:** 1859-06

**Authors:** 


### *Convention of Medical Teachers.*

### MORNING SESSION.

At a meeting of the last National Medical Convention, held in Washington City, it was resolved, that there should be a National Convention of the Teachers from the Medical Colleges in the United States, and that they should meet in the city of Louisville, Ky., the day before the meeting of the National Medical Convention. In accordance with this resolution, they met on Monday morning, May 2, 1859, at Mozart Hall.

After prayer by the Rev. J. H. Heywood, Dr. Dixi Crosby, Professor of Surgery in Dartmouth College, N. H., was called to the chair, and Dr. George C Blackman, of Ohio, appointed Secretary.

Dr. Crosby returned his thanks to the convention in a neat and appropriate speech.

Dr. D. F. Wright offered the following resolution :

*Resolved*, That all members of the Faculties of Medical Colleges, now present, shall be considered members of this convention; but that when more than one from the same College is present, but one of them shall cast the vote of that institution.

A substitute was offered by Dr. Baker, of Ohio, that a committee of three, on credentials, be appointed by the chair. This substitute for the original resolution was then carried, and the chair appointed the following gentlemen to serve on that committee : Dr. Shattuck, from Massachusetts, Dr. Haskins, from Tennessee, and Dr. Baker, from Ohio.

The convention then adjourned for thirty minutes, to allow the committee on credentials time to report. At the expiration of the time, the committee reported the following Colleges as represented, with the gentlemen as delegates :

*Dartmouth College, N. H.*—Prof. Dixi Crosby.

*Shelby Medical College, Tenn.*—Profs. E. B. Haskins, and D. F. Wright.

*Missouri Medical College*—Prof. J. H. McDowell.

*St. Louis Medical College*—Prof. N. L. Linton.

*Medical College of South Carolina*—Prof. H. R. Frost.

*Medical College of Georgia, Augusta*—Prof. H. F. Campbell.

*Medical Department of the University of Michigan*—Prof. Moses Gunn.

*University of Louisville, Medical Department*—L. P. Yandell and L. Powell.

*Cincinnati College of Medicine and Surgery*—Prof. H. Baker.

*Jefferson Medical College, Philadelphia*—Profs. Robly Dunglison, and Franklin Bache.

*Lind University of Chicago*—Prof. N. S. Davis.

*Oglethorpe Medical College, Georgia*—Prof. A. G. Thomas.

*Medical College of Ohio*—Prof. George C. Blackman.

*Kentucky School of Medicine*—Profs. M. Goldsmith, and Geo. W. Bayless.

*Iowa University*—Prof. McGugin.

*Medical College of Memphis*—Prof. H. R. Robards.

*Medical College of Virginia, Richmond*—Profs. B. R. Welford, and L. S. Joynes.

*Atlanta Medical College, Georgia*—Profs. J. G. Westmoreland, and John W. Jones.

*Medical Faculty of Harvard University at Boston*—Prof. Geo. C. Shattuck.

*Rush Medical College, Chicago, Ills.*—Profs. Daniel Brainard, and Joseph W. Freer.

*Western Reserve Medical College, Cleveland, Ohio*—Prof. G. C. C. Weber.

On motion, the report was received and the committee allowed to report further the names of delegates during the day.

The next business in order was the election of officers for the permanent organization of the convention.

On motion, the officers of the preliminary meeting were declared elected.

Prof. Crosby, in taking his seat as President, said, the convention had derived one advantage from the re-election of the present officers, as they were spared being inflicted with additional speeches of thanks. He concluded by saying that, unless changed by the convention, he had the authority to make rules for conducting business. He, therefore, ruled that no member shall speak longer than ten minutes, nor more than twice on the same subject.

A motion was offered by Dr. D. F. Wright, of Tenn., allowing all persons present from the different Medical Colleges to sit as members of this convention. This was amended by Dr. Davis, of Chicago, by adding that no College should be allowed more than one vote on any proposition. This was carried.

Dr. Davis, of Ills., offered a resolution authorizing the appointment of a committee of five, as a business committee. This being carried, the President appointed the following gentlemen: "Drs. Davis, of Ills., Gunn, of Michigan, Frost, of S. C., Shattuck, of Mass., and Yandell, of Louisville, Ky.

The convention then adjourned for thirty minutes, to allow the committee time to prepare business.

At the expiration of the time, the committee reported six resolutions, which were accepted from the committee, and were, on motion, brought up in order. The following are the resolutions:

*Resolved,* That this convention recognize the great advantages to be derived from the action of the American Medical Association in prescribing the terms and conditions on which medical degrees shall be conferred and licenses to practice medicine shall be granted, and that any expression of opinion as to methods or periods of instruction from the American Medical Association should be received with deference and respect, and that all pains should be taken to enforce any rules or regulations recommended by that body

*Resolved,* That this convention earnestly recommends the American Medical Association to adopt such measures as will secure the efficient practical enforcement of the standard of the preliminary education, adopted at its organization in May, 1847, and that the Medical Colleges will cheerfully receive and record the certificates alluded to in said standard, whenever the profession generally, and the preceptors, will see that students are properly supplied with them.

*Resolved*, That no Medical College should allow any term of practice to be a substitute for one course of lectures in the requisitions for graduation.

*Resolved*, That hospital clinical instruction constitutes a necessary part of medical education, and that every candidate for the degree of Doctor of Medicine should be required to have attended such instruction regularly for a period of not less than *five months*, during the last year of his period of pupilage.

*Resolved*, That every Medical College should rigidly enforce the rule requiring three full years of medical study before graduation, and that the diploma of no Medical College shall be recognized which is known to violate this rule

The first resolution created some degree of excitement, and provoked considerable debate, when the convention adjourned until three o'clock.

AFTERNOON SESSION.

The debate still continued on the first resolution, as reported in the forenoon. Dr. Bayless, who had the floor at the time of adjournment, offered the following amendments—to substitute, in the fourth line, the word "recommended" for "prescribe," and all after the words "deference and respect" be stricken out.

The debate on this resolution still continued warm, most of the gentlemen participating, when Dr. Joynes offered the following as a substitute :

WHEREAS, It appears that a large portion of the Medical Colleges of the United States are unrepresented in this convention, that no changes in the present system of education can be effected unless adopted by the schools generally.

*Resolved*, That it is inexpedient at this time to take any action upon the propositions contained in the report presented by the special Committee on Medical Education at the last meeting of the American Medical Association.

*Resolved*, That with the view of obtaining a more general union in counsel and action upon this important subject, this convention do now adjourn to meet again on the day preceding the next annual meeting of the Medical Association, and at the place which may be agreed upon for such meeting, and that the Medical Colleges in the United States be requested to appoint each a delegate to such adjourned meeting of this convention.

An amendment was offered by Dr. Wright, by adding another resolution to the effect that a committee be appointed to examine the different propositions offered. The vote of the Colleges being called on this resolution, the vote stood ten for the substitute and nine against.

The following gentlemen were appointed by the chair to serve on that committee : Dr. L. P. Yandell, of Louisville ; Dr. G. Shattuck, of Massachusetts ; Dr. G. C. Blackman, of Ohio ; Dr. H. F. Campbell, of Georgia, and Dr. M. Gunn, of Michigan.

The meeting was then adjourned.

